# Effect of Varying Stiffness and Functionalization on the Interfacial Failure Behavior of Isotactic Polypropylene on Hydroxylated γ-Al_2_O_3_ by MD Simulation

**DOI:** 10.1021/acsami.2c19593

**Published:** 2023-01-20

**Authors:** Yoshitake Suganuma, James A. Elliott

**Affiliations:** Department of Materials Science and Metallurgy, University of Cambridge, 27 Charles Babbage Rd, CB3 0FSCambridge, United Kingdom

**Keywords:** isotactic polypropylene, aluminum oxide, polymer−metal joint, tensile strength, molecular dynamics simulation, Young’s modulus

## Abstract

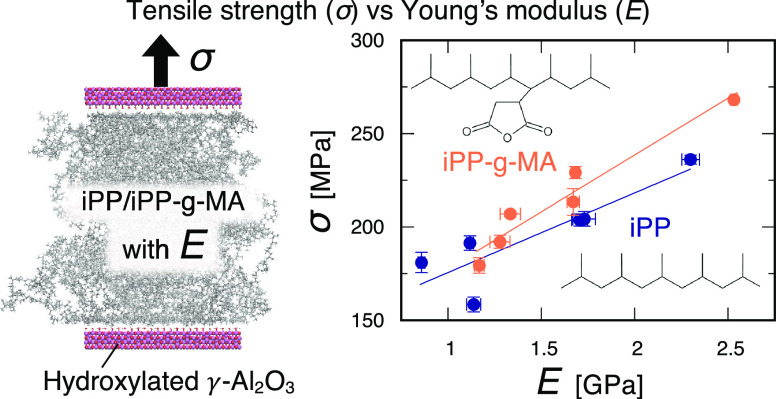

This study focuses on polymer–metal joints consisting of isotactic polypropylene (iPP) or iPP grafted with maleic anhydride (iPP-g-MA) and hydroxylated γ-Al_2_O_3_, which is a model for an oxidized aluminum surface, and investigates the contributions of the Young’s moduli of iPP and iPP-g-MA and chemical functionality (MA groups) in iPP-g-MA to the interfacial failure behaviors using the molecular dynamics (MD) simulation method. First, our calculations demonstrated that the tensile strength observed in interfacial failures of the joints increases as Young’s modulus of the polymer in the joints increases. This is because a higher stiffness makes it harder for a void to form within the polymer matrix under the applied tensile strain and to reach the interface. Second, in iPP-g-MA−γ-Al_2_O_3_ joints, MA groups work more effectively to improve the interfacial strength as the Young’s modulus of the polymer in the joints increases. For iPP-g-MA with a lower Young’s modulus, the polymer molecules are pulled off the surface in a peel mode with increasing normal strain due to their greater flexibility. This results in a gradual removal of the MA groups and thus reduces their contribution. Meanwhile, for a higher Young’s modulus, iPP-g-MA molecules at the interface are removed in a tensile mode because of their increased stiffness. This leads to more MA groups required to be detached from the surface at the same time to cause interfacial failure, thus increasing the contributions of the MA groups.

## Introduction

The transportation sector, including automotive, aviation, and maritime industries, is one of the largest contributors to greenhouse gas emissions around the world, accounting for 14% of global green gas emissions in 2018, a figure which is still currently increasing year-on-year.^1^ To mitigate this impact and also to meet customers’ needs for more environmentally friendly and more low-cost products or services, these industries have been making strenuous efforts to improve fuel efficiency. One commonly used strategy to achieve higher fuel efficiency is weight reduction in vehicle architectures by replacing metal parts with ones made of plastic. According to a recent study,^2^ in on-highway passenger vehicles, a 10% weight reduction leads to an improvement in fuel economy of between 6 and 8%. Furthermore, it was reported that a 20% weight reduction was achieved for the Boeing 787 aircraft by incorporating 50% carbon fiber composite by weight instead of aluminum, which resulted in a 10–12% improvement in fuel efficiency. These improvements rely on multimaterial structures in which polymers and metals are required to be strongly bonded together. However, designing appropriate bonding processes for polymers and metals is often challenging due to the many parameters affecting their interfacial properties, such as the mechanical properties and functional groups of metals and polymers, and microstructures of the metal surfaces.^3,4^ In addition to the properties of polymers and metals, the bonding processes themselves usually involve many parameters, including curing time and temperature for polymers and surface treatment for polymer or metal surfaces, which also need to be optimized to achieve a strong and reliable bond.^5^ However, as yet, there is not a full understanding of the contributions of these factors, which makes designing joining processes difficult.

To address this issue, many studies have investigated interfacial phenomena using computational techniques, such as density functional theory (DFT) and molecular dynamics (MD) calculations. Some have revealed that functional groups in polymers, such as amine, epoxy, and methacrylate groups, improve the interaction with metal/metal oxide surfaces, leading to a better bonding.^6−9^ Others examine the contributions of microstructures on metal/metal oxide surfaces. For example, Iwamoto studied an epoxy–copper oxide interface, revealing that roughness of the copper oxide surface increases the involvement of the polymer at the interface when a shear or tensile stress is applied, and that a higher contribution of the roughness is observed in a shear mode.^10^ These works show that simulation techniques can provide important information to choose suitable functional groups in polymers and to build an effective roughness on metal surfaces to improve interfacial properties in polymer–metal joints. Nevertheless, some parameters which may influence interfacial properties, such as the stiffness and ductility of polymers, have not yet been examined in detail.

To make clearer the bonding mechanisms between metals and polymers, this work investigates the contributions of Young’s modulus and chemical functionality of a polymer to the interfacial failure behaviors using MD calculations. For the combinations of brittle coatings on ductile substrates, such as ceramic coatings on metal surfaces, there are some experimental studies reporting that the interfacial shear strength is significantly affected by Young’s modulus of brittle coatings using specially designed samples and mechanical tests.^11−13^ For polymers, some works have discussed the influence of Young’s modulus of polymers on cohesive failure behaviors in polymer–metal joints by conventional tensile tests.^14^ However, there is still a poor understanding of its contribution to the interfacial failure behaviors. Therefore, this work aims at evaluating how Young’s modulus of a polymer affects the interfacial failure behavior for a polymer–metal joint in conventional tensile tests using MD calculations. For the polymer, we chose amorphous isotactic polypropylene (iPP), and for the metal, we chose aluminum because they are both commonly used lightweight materials in automobile industries. Since the aluminum surface is always oxidized under normal operating conditions, the surface of hydroxylated γ-Al_2_O_3_ (the most commonly occurring oxide surface) was built as a substrate for joining with iPP.

In addition to the contribution of the stiffness of iPP, our calculations investigate the different contributions of interfacial interactions depending on Young’s modulus. When joining iPP, different functional groups are often introduced to improve its interactions with metals and other dissimilar materials.^15−17^ One of the most commonly used techniques to modify an iPP is grafting of maleic anhydride.^17^ There are many studies reporting that iPP grafted with maleic anhydride (iPP-g-MA) shows higher interfacial strength with metals than iPP.^18−20^ Some of these also examine the optimized concentration of the MA group in iPP-g-MA to maximize the interfacial properties.^18^ However, the influence of Young’s modulus on modifying the contribution of higher interfacial interactions still remains unclear. To address this issue, MD calculations were performed using iPP-g-MA structures with different stiffnesses.

In summary, this work studies how Young’s moduli of iPP and iPP-g-MA affect the interfacial failure behaviors with the surface of hydroxylated γ-Al_2_O_3_ and further investigates how Young’s modulus of iPP-g-MA influences the contribution of its higher interfacial interaction to the interfacial failure behaviors. Hereafter, we summarize the simulation methods, including construction of the bulk amorphous iPP and sandwich-structured models with γ-Al_2_O_3_, and the introduction of “entanglement points” to modify the polymer stiffness. The protocol for conducting mechanical tests via molecular simulation is also described. Then, we discuss the results of stress–strain curves from iPP and iPP-g-MA with different stiffnesses and rationalize the findings in terms of two different failure mechanisms, which control the strength of polymer–metal interface depending on stiffness polymer matrix.

## Simulation Method

### Building Bulk Amorphous Polymers, and Sandwich-Structured Models

In the present work, the bulk structures of amorphous iPP and iPP-g-MA were prepared to evaluate their bulk properties. Then, using these, sandwich-structured models in which an iPP or iPP-g-MA was placed between two hydroxylated aluminum oxide surfaces were built to investigate the interfacial failure behaviors.

#### Bulk Structure of Amorphous iPP

To obtain a representative model for amorphous iPP, 100 rotationally disordered iPP chains each consisting of 100 repeat units were independently generated at 298 K by using Amorphous Cell module in BIOVIA’s Materials Studio 2019^21^ such that 25 iPP molecules were packed with an experimental density for amorphous iPP of 0.850 g cm^–3^.^22^ Then, their energies were compared and the structure with the lowest configurational energy was chosen as an initial structure of iPP. This was further relaxed by an MD simulation employing an NpT ensemble for 5 ns with the cutoff distance of 18.5 Å and a timestep of 1.0 fs. Hereinafter, MD simulations were carried out using the open-source software package LAMMPS,^23^ and Nosé–Hoover forms of thermostat and barostat were employed for NVT and NpT ensembles, respectively.^24^ The PCFF force field^25^ was used in these calculations.

#### Bulk Structure of Amorphous iPP-g-MA

For the bulk structure of iPP-g-MA, 25 iPP-g-MA molecules each consisting of 100 repeat units were prepared such that each molecule was randomly grafted with three MA groups. This corresponds to a grafting ratio of 3.2 wt %, which is slightly higher than around 1 wt % in commercially available iPP-g-MA.^19^ Then, all of the five-membered rings in the MA groups were manually hydrolyzed because they easily open to form two carboxylic acids under ambient humidity and via absorbed water on metal surfaces.^20^ Using these iPP-g-MA molecules, 100 amorphous structures of iPP-g-MA were built at 298 K, similarly to the procedure for nongrafted iPP. After that, a single structure with the lowest energy was selected as an initial structure of iPP-g-MA and relaxed by an MD simulation using NPT ensemble for 5 ns.

#### Sandwich-Structured Models

**Figure 1:**
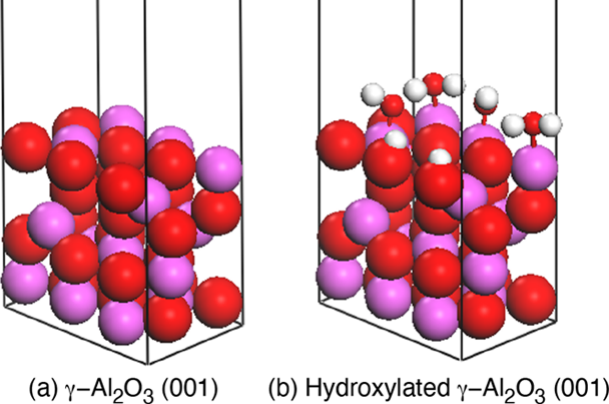
Surface models of (a) γ-Al_2_O_3_ (001) and (b) hydroxylated γ-Al_2_O_3_ (001). Aluminum, oxygen, and hydrogen atoms are shown in pink, red, and white, respectively.

For the surfaces, γ-Al_2_O_3_ was chosen because it is the most thermodynamically stable crystal form in the thin surface layer of native alumina.^26,27^ Furthermore, the γ-Al_2_O_3_ surface is readily hydroxylated in the presence of humid air.^28,29^ Therefore, a hydroxylated γ-Al_2_O_3_ surface was prepared as a model of a hydroxylated aluminum oxide surface according to a theoretical work using DFT calculations by Digne et al.^30^ First, the bulk crystal structure of γ-Al_2_O_3_ was obtained from their study. Then, it was cleaved along the (001) crystallographic plane to build the γ-Al_2_O_3_ (001) surface as shown in Figure 1a. At the start of the hydroxylation, water molecules are absorbed on aluminum atoms on the surface. After that, they are dissociated to hydroxy groups and protons to protonate the nearest oxygen atoms. Digne et al. showed that the hydroxylated γ-Al_2_O_3_ (001) surface where all of four aluminum atoms exposed on the cleaved surface in Figure 1a are hydroxylated is the most stable. This means that four of the six oxygen atoms on the surface should be protonated. Following this work, first, all of the four aluminum atoms were manually hydroxylated and four of the six oxygen atoms were selected to be protonated to obtain _6_C_4_ (=15) initial structures for the hydroxylated γ-Al_2_O_3_ (001) surface. They were subjected to optimizations by DFT calculations, and then their energies were compared to choose the one with the lowest energy. On the selected surface, two hydrogen atoms protonating oxygen atoms on the surface were closer to oxygen atoms in the nearest hydroxy groups than to the oxygen atoms they are bonded to. Hence, bonds between the hydrogen atoms and their protonating oxygen atoms were manually cut, and they were bonded to the nearest oxygen atoms in the hydroxy groups. The final hydroxylated γ-Al_2_O_3_ (001) surface is shown in Figure 1b. The charges of the atoms in the surface were calculated by Hirshfeld population analysis for the following MD simulations.^31^ The DFT calculations were performed by using CASTEP software package (version 18.1) in BIOVIA’s Materials Studio 2019.^32,33^ For the optimizations, the Perdew–Burke–Ernzerhof (PBE) form of the generalized gradient approximation (GGA) was employed and an OTFG ultrasoft pseudopotential was used.^34^ The kinetic energy cutoff was set to 630 eV. A 3 × 2 × 1 Monkhorst–Pack mesh was used for k-point sampling.^35^

**Figure 2:**
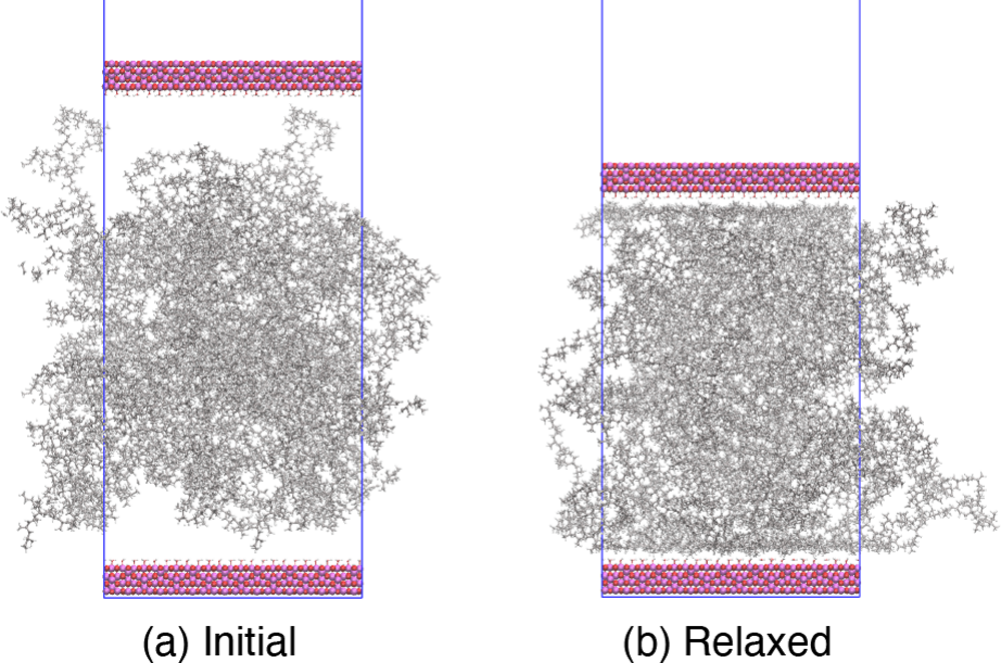
(a) Initial and (b) “relaxed” sandwich-structured models of iPP.

To build the sandwich-structured models of iPP and iPP-g-MA, the surface was expanded to a size of 44.7 × 67.3 Å^2^. Then, the nanostructures of iPP and iPP-g-MA with the same *x*- and *y*- widths as the surface and a thickness of 100 Å were created from the bulk structures of iPP and iPP-g-MA obtained above and placed onto the surface. Next, the surface was duplicated, inverted, and placed above the iPP or iPP-g-MA to build the initial structures of the sandwich-structured models. They were then relaxed by a four-step MD simulation protocol. In the first three steps, a pressure of 1 atm was applied onto the upper surface while it was allowed to move only along the *z*-axis. First, an MD simulation was performed for 2 ns at 498 K, which is above the melting temperatures of iPP and iPP-g-MA. Second, the sandwich-structured models were cooled to 298 K over 2 ns followed by the third MD simulation at 298 K for 2 ns. Finally, the pressure onto the upper surface was removed and an MD simulation was conducted at 298 K for 1 ns while all of the atoms in the both surfaces were constrained to their last positions in the previous MD simulation. The initial and final (“relaxed”) sandwich-structured models of iPP are shown in Figure 2.

### Introduction of “Entanglement Points”

In this work, iPP and iPP-g-MA structures with different Young’s moduli were prepared by introducing “entanglement points” that cannot be disentangled and behave like crosslinking points during mechanical tests. This is based on experimental observations that physical entanglement points in iPP are not disentangled during tensile tests.^36^ Those with different physical entanglement densities are expected to have different Young’s moduli despite having the same chemical structures.

To introduce “entanglements” into the bulk structures of iPP and iPP-g-MA and those in the sandwich-structured models, backbone carbon atoms in iPP or iPP-g-MA were randomly chosen depending on the number of entanglement points to be targeted. Then, the closest backbone carbon atom to each of them was found from another molecule to make a pair. In mechanical tests in MD simulations, these pairs were independently treated as rigid bodies. This means that forces and torques exerted on atoms in a rigid body are summed to calculate the total force and torque on the body at each timestep. Then, the velocities and orientations of the atoms in each body are updated such that the movement of the body is consistent with the total force and torque. The entanglement points were introduced into the bulk structures of iPP and iPP-g-MA and those in the sandwich-structured models every 20 (EP1/20), 10 (EP1/10), 5 (EP1/5), 4 (EP1/4), 3 (EP1/3), and 2 (EP1/2) repeat units on average. The ratios between molecular weight (*M*_w_) and entanglement molecular weight (*M*_e_), *M*_w_/*M*_e_, are evaluated as between 2.5 and 25, which are not very different from experimentally obtained values of 15 or more.^37^ The densities of the entanglement points range from 2.2 × 10^20^ to 2.9 × 10^21^ cm^–3^. These values are comparable to cross-link densities in real materials such as chemically cross-linked polyolefins and polyolefin elastomers.^38,39^ The models with entanglement points were further validated by evaluating their stress–strain curves, as discussed later.

### Mechanical Tests in MD Simulations

The stress–strain curves for the bulk structures of iPPs and iPP-g-MAs with different numbers of the entanglement points, and their sandwich-structured models were evaluated to determine the properties of the bulk structures and failure behaviors in the sandwich-structured-models. For the bulk structures of iPPs and iPP-g-MAs, three MD simulations were performed to deform them along the *x*-, *y*-, and *z*-axes with an engineering strain rate of 1.0 × 10^10^ s^–1^. The deformation rate is much higher than those usually employed in experiments. However, an experimental work has demonstrated that this does not significantly change the trend of mechanical properties such as yield stress observed in amorphous polymers at different temperatures, which corresponds to polymers with different Young’s moduli.^40,41^ This implies that even in mechanical tests using a high strain rate, mechanical properties are still comparable and their trend can be meaningfully discussed. During the simulations with the deformations imposed along the *x*-, *y*-, and *z*-axes, the *xx*-, *yy*-, and *zz*-components of the stress tensors were recorded, respectively, every 100 steps to obtain three stress–strain curves. Then, they were averaged over the three directions. For the sandwich-structured models, the upper surface was moved along the *z*-axis such that an iPP or iPP-g-MA between the surfaces was deformed with the same engineering strain rate as used for the bulk structures. The forces exerted on the upper and lower surfaces were evaluated every 100 steps. Then, they were divided by the surface area and averaged over the two surfaces to obtain a stress–strain curve.

## Results and Discussion

**Figure 3:**
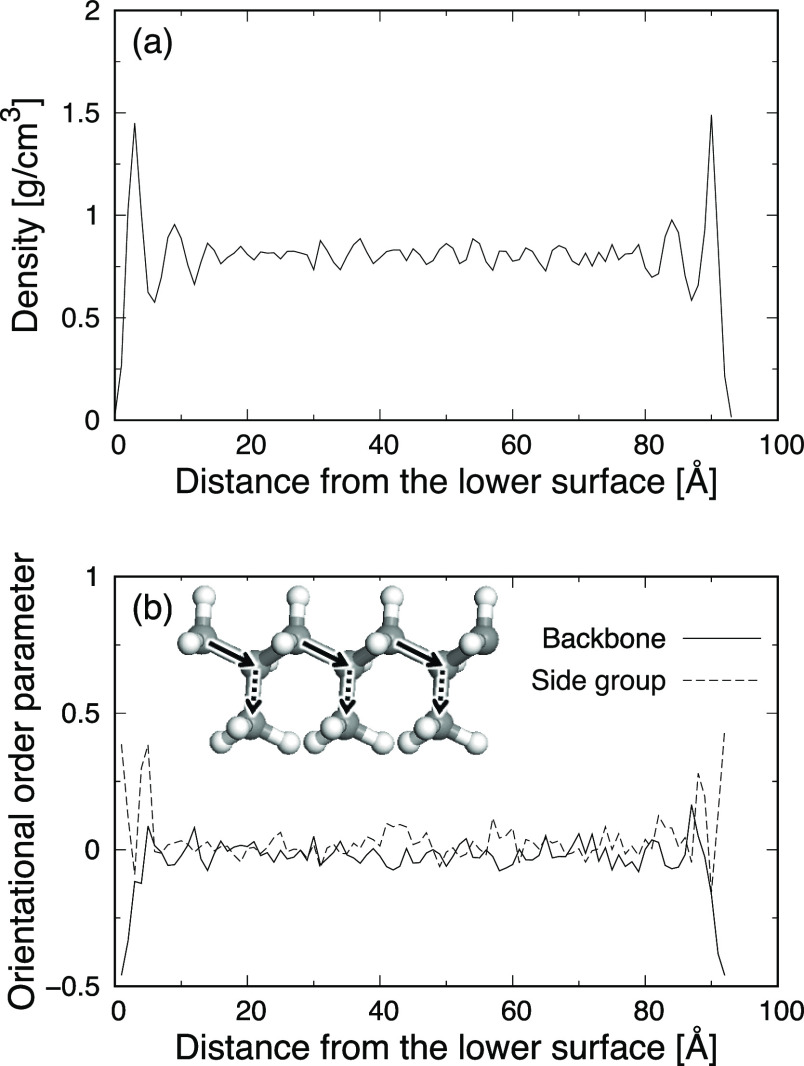
Profiles of (a) density of iPP and (b) orientational order parameters as a function of the distance from the lower surface for the sandwich-structured model of iPP.

First, to validate the sandwich-structured models before the entanglement points were introduced, the profiles of the density of iPP or iPP-g-MA and orientational order parameters between the surfaces were evaluated using the last MD simulation in the four-step MD simulation protocol. For the orientational order parameters, two vectors were defined in iPP or iPP-g-MA molecules: one vector between 1 and 2 neighboring backbone carbon atoms and the other between carbon atoms in the backbone and side group (vectors shown with solid and dotted lines, respectively, in Figure 3b). The orientational order of these vectors can be characterized by an orientational order parameter *P* given by
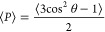
1where θ is the angle between the vector in iPP or iPP-g-MA molecules and the surface normal. The positions of these vectors were set to their geometry center of masses.

Figure 3 shows the profiles of the density of iPP and orientational order parameters as a function of the distance from the lower surface for the sandwich-structured model of iPP. The left and right edges in these profiles at zero distance and around 93 Å correspond to the positions of the lower and upper surfaces, respectively. The density profile indicates that the density fluctuates around 0.81 g cm^–3^ in the bulk region between around 30 and 70 Å, while a few layers of dense and sparse areas are observed near the surfaces. On the other hand, the orientational order parameter for the backbone vector takes the minimum values of around −0.5 at the surfaces. This signifies that the backbone chains are absorbed on the surfaces and, therefore, are more likely to be aligned in plane. This is why the side-group vector shows the value of around 0.4, which corresponds to their normal orientations, and dense areas were seen at the surfaces in the profile of the density. The profiles of the density and orientational order parameters in the sandwich-structured model of iPP-g-MA also show these features caused by the absorption on the surfaces (see Figure S1 in Supporting Information). This is consistent with other computational works on interfaces between a polymer and a metal or metal oxide surface.^42^

**Figure 4:**
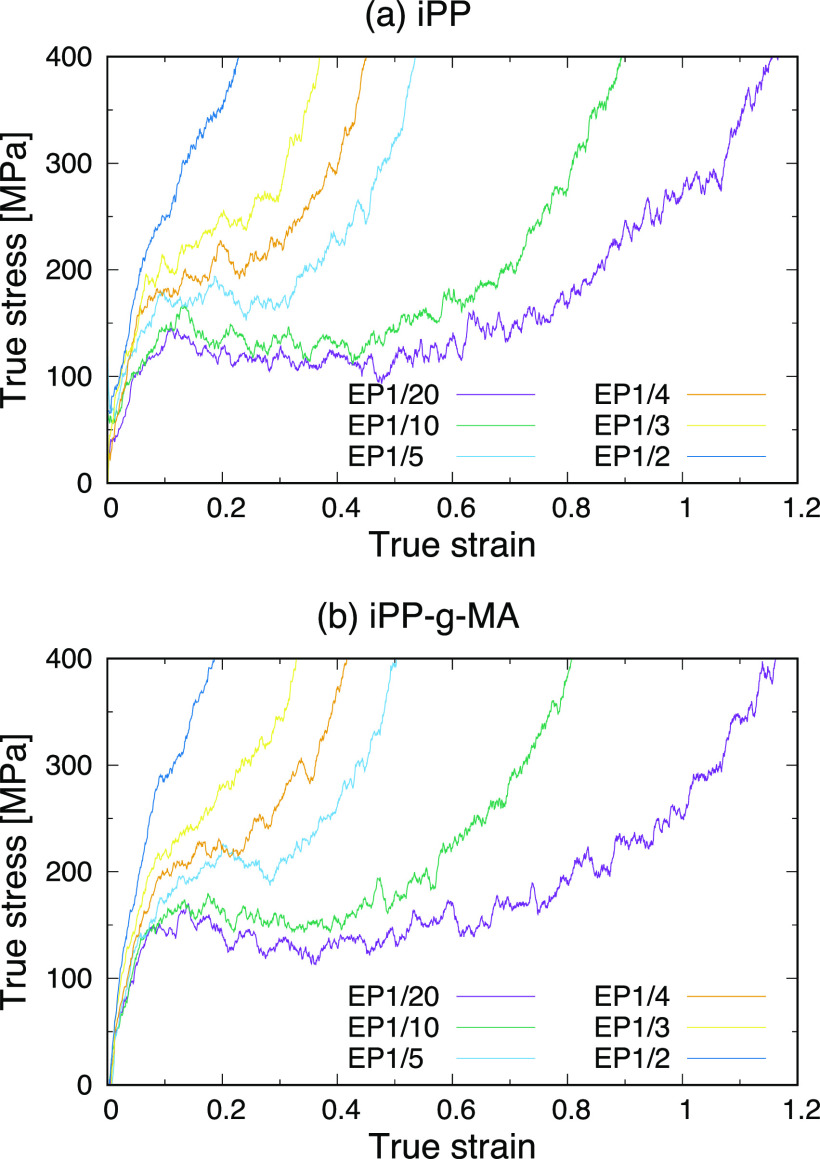
Stress–strain curves of (a) iPP and (b) iPP-g-MA structures with different numbers of entanglement points.

Next, to demonstrate how the “entanglement points” introduced into the bulk structures of iPP and iPP-g-MA affect their properties, Figure 4 shows their stress–strain curves. The behaviors in these curves are in good agreement with those observed in real materials.^43^ Assuming that all of the iPP and iPP-g-MA structures fail at the same stress, those with the lower number of entanglement points show a more gradual increase in the stress after the yield point, failing at a larger stress. This is a similar behavior to soft, tough materials like rubbers. As the number of entanglement point increases, the stress value rises more rapidly after the yield point and the extension at failure gets smaller. These trends can be seen in the stress–strain curves of hard, tough materials such as polycarbonate, and even more brittle polymers including nylon, rather than rubbers. These features, consistent with experimental observations, indicate that introducing different numbers of “entanglement points” provides reasonable materials with a range of different properties.

**Figure 5:**
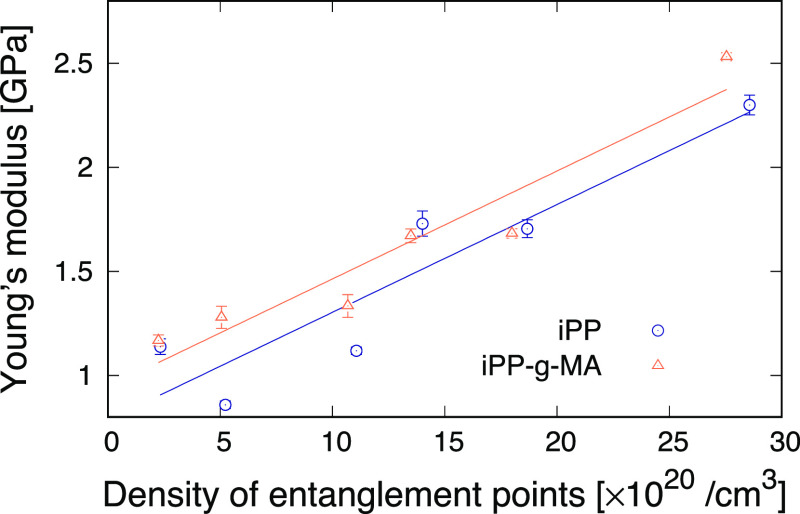
Young’s moduli of iPP and iPP-g-MA as a function of the density of entanglement points.

Using these stress–strain curves, Young’s moduli of iPP and iPP-g-MA structures were evaluated. A best-fit line for the data at true strains of between 0.02 and 0.1 where the stress value increases linearly with the strain value was obtained by a linear regression, and Young’s modulus was determined from its slope. Figure 5 shows Young’s modulus as a function of the density of the entanglement points for iPP and iPP-g-MA. This suggests that a larger density of the entanglement points provides a higher Young’s modulus. This is consistent with experimental works which also show a linear variation of stiffness with cross-link density.^20,44,45^

**Figure 6:**
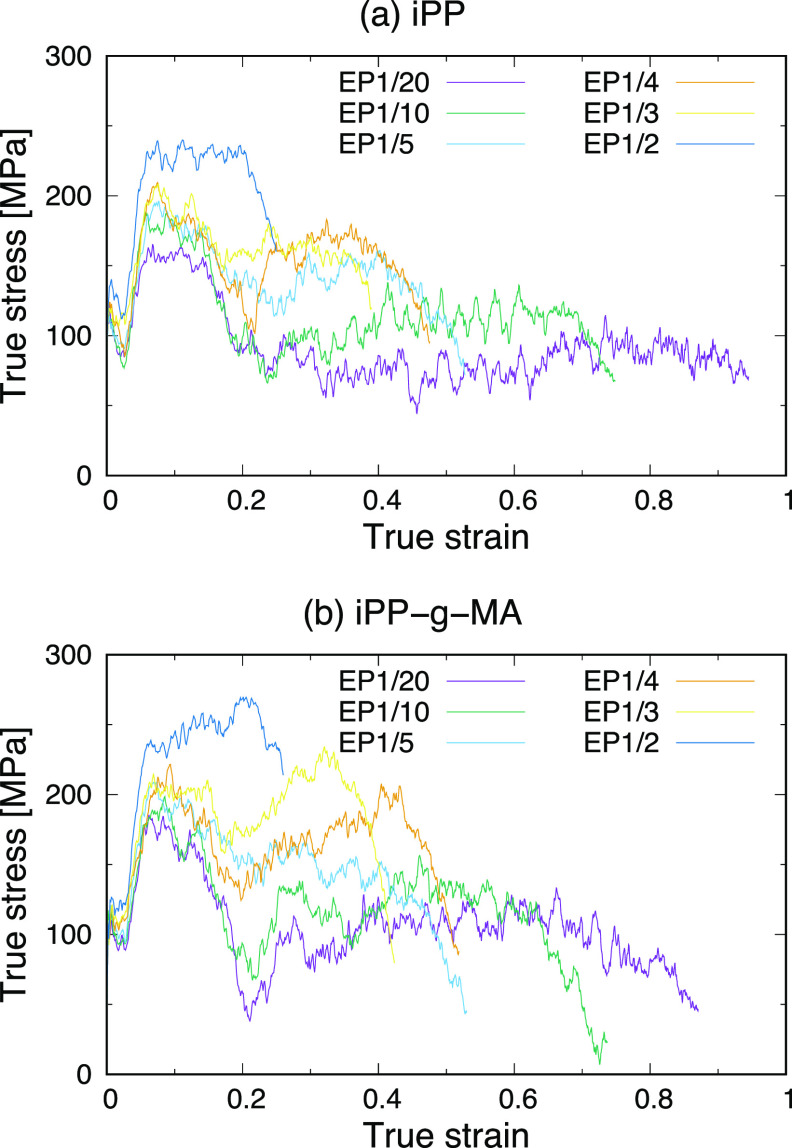
Stress–strain curves of sandwich models of (a) iPPs and (b) iPP-g-MAs with different number of entanglement points.

**Figure 7:**
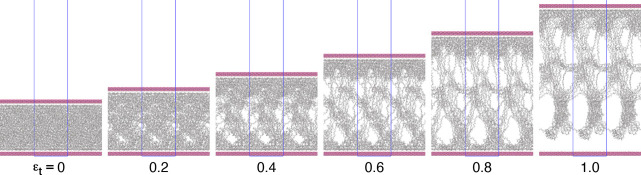
Snapshots of the sandwich-structured model of iPP-EP1/20 at true strains (ϵ_*t*_) of 0.0, 0.2, 0.4, 0.6, 0.8, and 1.0, respectively, during a tensile test in an MD simulation.

Figure 6 shows the stress–strain curves of the sandwich-structured models of iPP and iPP-g-MA in order to investigate their failure behaviors. In all of these curves, yield points are observed at a true strain of just under 0.1. After that, the sandwich-structured models with a lower number of entanglement points failed at larger strains. To show how they fail, Figure 7 shows snapshots of the sandwich-structured model of iPP-EP1/20, as an example, at true strains of 0.0, 0.2, 0.4, 0.6, 0.8, and 1.0 during a tensile test. This indicates that, first, some voids are generated within the iPP, then grow, and reach the interface, finally resulting in an interfacial failure. All of the sandwich-structured models shown in Figure 6 fail by the same mechanism: generation of voids within an iPP or iPP-g-MA, their development, and an interfacial failure caused by their reaching the interface.

**Figure 8:**
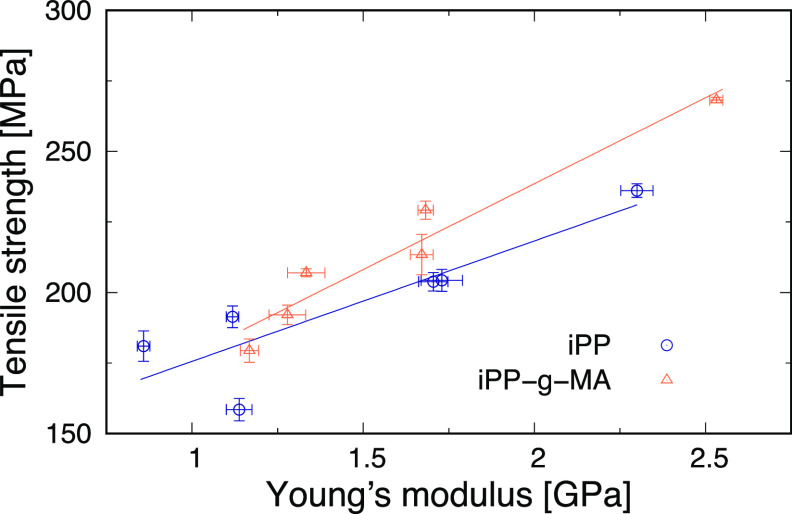
Relationship between Young’s moduli of the bulk structures and tensile strengths of the sandwich-structured models for iPP and iPP-g-MA.

Next, tensile strengths in the sandwich-structured models were evaluated and related to Young’s moduli of iPP and iPP-g-MA structures to discuss their relationships. In Figure 6, the tensile strengths of the sandwich-structured models of iPP and iPP-g-MA structures were calculated as an average over the seven data points before and after the maximum stress value. Then, Figure 8 shows the relationships between Young’s moduli of the bulk structures and tensile strengths of the sandwich-structured models for iPP and iPP-g-MA. In both the iPP and iPP-g-MA, the tensile strength observed in the sandwich-structured models increases as Young’s moduli of iPP and iPP-g-MA get larger. As mentioned above, all of the sandwich-structured models fail at the interface. Therefore, the Young’s moduli of iPP and iPP-g-MA significantly affect the tensile strengths observed in the interfacial failures. This is because the interfacial failures start with void formation within the iPP or iPP-g-MA, and the higher Young’s modulus makes it more difficult for them to initiate.

Then, the influence of interfacial interactions on the interfacial failure behaviors was investigated. Using the sandwich-structured models of iPP and iPP-g-MA before the introduction of the entanglement points, the interfacial energies were evaluated by
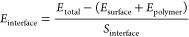
2where *E*_total_ is the total energy of the system, *E*_surface_ is the energy of the surfaces without polymer, *E*_polymer_ is the energy of iPP or iPP-g-MA without the surfaces, and *S*_interface_ is the interfacial area between the surfaces and iPP or iPP-g-MA. The interfacial area was set to the surface area of the surfaces. The interfacial energies were averaged over the last 1 ns in the last MD simulations. The interfacial energies were calculated as 121.2 and 140.9 mJ m^–2^ for the sandwich-structured models of iPP and iPP-g-MA, respectively. This is consistent with experimental results that the MA groups grafted into iPP enhance interfacial strength with metal or metal oxide surfaces due to the stronger interactions of the MA groups with the surfaces.^11,12^

To examine the influence of the higher interfacial interaction of the MA groups in our calculations, the failure behaviors in the tensile tests using the sandwich-structured models were further investigated. In the stress–strain curves of the sandwich-structured models in Figure 6, two maximal stress values are clearly observed except for the EP1/2 models of iPP and iPP-g-MA. One of the maxima can be seen at the true strain of around 0.1. At this point, voids start to generate within the iPP or iPP-g-MA models between the surfaces and there are no significant defects at the interfaces as shown in Figure 7. This means that the maximum corresponds to the yield point of iPP or iPP-g-MA between the surfaces and, therefore, the stress value at this point was defined as a yield stress in the sandwich-structured models. After the yield point, the stress decreases (apart from the EP1/2 models of iPP and iPP-g-MA) followed by a rise, while the iPP or iPP-g-MA chains are aligned along the applied strain direction. Then, it reaches the second maximal value before reducing again. At the true strain of the second maximal value, the interface starts to fail. This means that it corresponds to the interfacial strength in a sandwich-structured model. For EP1/2 models of iPP and iPP-g-MA, although there are no significant maximal stress values, a similar phenomenon can be observed in their stress–strain curves. They show an inflection point at a true strain of around 0.1, which corresponds to the yield point of an iPP or iPP-g-MA between the surfaces. Next, the stress drops abruptly at a true strain of around 0.2, at which point the sandwich-structured models start to fail at the interface.

**Figure 9:**
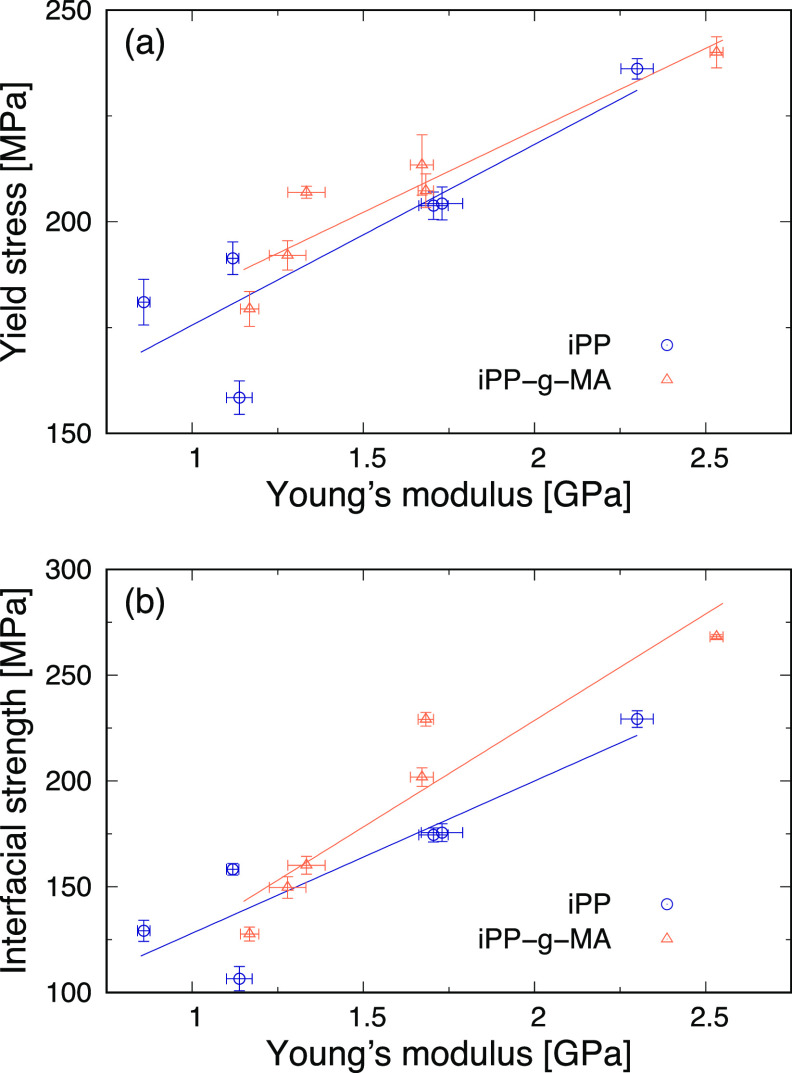
(a) Yield stress and (b) interfacial strength observed in the sandwich-structured models of iPP and iPP-g-MA as a function of Young’s modulus.

To examine how these two characteristic values, yield stress and interfacial strength, are affected by Young’s modulus and interfacial interaction, they were evaluated in the stress–strain curves of sandwich-structured models (Figure 6). The yield stress was defined as the maximum value in the region of strain less than 0.1. This is because iPP or iPP-g-MA yields between the surfaces in this region and a maximal value was observed after the yield except for EP1/2 models of PP and iPP-g-MA, in which there are not clear maximal value in the region. For the evaluation of yield stress of EP1/2 models, first, the left edge of the yield region was set to a true strain of 0.02. Then, its right edge was moved from a true strain of 0.03 one data point at a time while the determination coefficient for a least-squares linear fit to the region was monitored. The true strain that gave the maximum value of the determination coefficient was defined as the yield point. The stress values were averaged over the seven data points before and after the yield point to obtain a yield stress, which is an average of the 15 data points in total. Meanwhile, the interfacial strength was calculated by averaging over the seven stress values before and after the maximum value after yielding. Figure 9 shows the yield stress and interfacial strength as a function of Young’s modulus for iPP and iPP-g-MA. This reveals that the yield stress observed in the sandwich-structured models increases as Young’s modulus of iPP or iPP-g-MA increases. Additionally, the slopes for iPP and iPP-g-MA are almost the same. This is because these stress values are observed at the yield points when iPP and iPP-g-MA yield in the sandwich-structured models. Thus, they are mostly affected by Young’s moduli of iPP and iPP-g-MA structures and are not significantly influenced by their interfacial interactions. On the other hand, the interfacial strength also rises with increasing Young’s modulus. However, although the interfacial strengths of iPP and iPP-g-MA are almost overlapping each other at a Young’s modulus less than 1.5 GPa, those of iPP-g-MA are larger than those of iPP at a larger Young’s modulus, leading to a greater slope of iPP-g-MA. This implies that the interfacial interactions affect the interfacial strength more significantly at a larger Young’s modulus than at a lower Young’s modulus. These different contributions of the higher interfacial interaction of the MA groups to the interfacial strength are caused by the differences in the failure mode depending on Young’s modulus. At a lower Young’s modulus, which corresponds to the lower number of entanglement points, iPP or iPP-g-MA molecules have fewer fixed points, which act as pulling points in tensile tests. This leads the molecules to be removed from the surfaces in a peel mode. In a peel mode, MA groups in iPP-g-MA are more likely to be peeled from the surface one by one. This results in a reduced contribution of the higher interactions of MA groups. On the other hand, at a larger Young’s modulus, the molecules are detached in more like a tensile mode due to a higher rigidity of structure caused by the larger number of “entanglement points.” In the tensile mode, more MA groups are pulled and removed from the surfaces at the same time. Therefore, they affect the interfacial strength more significantly. The difference in the contributions of the higher interfacial interaction of MA groups between peel and tensile modes is demonstrated in the Supporting Information (see Figure S2).

In our models, we changed Young’s moduli of the polymers by changing the number of entanglement points. This affects the network structure of the polymer between the surfaces, which might also contribute to the failure behaviors as well as Young’s modulus. Especially in the interfacial strengths shown in Figure 9b, the influence of the network structure should be discussed because interfacial strengths are observed in a plastic region instead of in an elastic region where Young’s modulus is determined. However, as discussed above, even in interfacial failures, a greater stiffness of the polymer changes the failure modes at the interfaces from a peel-like mode to a tensile-like mode, leading to an increase of the interfacial strength. From the perspective of this mechanism, although other properties such as the topology might result in different contributions of the stiffness to the tensile strength, Young’s modulus is an appropriate parameter affecting the tensile strength (see Figures S3 and S4 in Supporting Information).

Comparing the yield stress in Figure 9a with the interfacial strength in Figure 9b, larger values were observed as tensile strengths in Figure 8. In the sandwich-structured models of iPPs, the yield stress is always higher than the interfacial strength due to the poor interfacial interaction. As a result, the tensile strengths in Figure 8 correspond to the yield stresses of iPPs in Figure 9a, being affected mainly by its Young’s modulus. For iPP-g-MA models with a lower Young’s modulus, the higher interfacial interaction of MA groups does not significantly contribute to the tensile strength due to the peel-mode failure of iPP-g-MA molecules at the interface. Hence, as with for iPPs, the yield stresses of iPP-g-MAs in Figure 9a are observed as the tensile strengths in Figure 8, and Young’s modulus is the most influential parameter on the tensile strength. Meanwhile, at a larger Young’s modulus, iPP-g-MA molecules are detached from the surfaces in a tensile mode, leading the greater contribution of the interfacial interactions. This makes the interfacial strength larger than the yield stress in Figure 9a,b and to be observed as the tensile strength in Figure 8. This is why the tensile strengths observed in interfacial failures are affected by Young’s modulus and the slope of iPP-g-MA in Figure 8 is also larger than that of iPP.

In our calculations, the interfaces in the sandwich-structured models were sufficiently strong for the polymer between the surfaces to yield. This resulted in the differences in the yield stress and interfacial strength, either of which corresponds to the tensile strength. However, if the interfaces are too weak for the polymer between the surfaces to reach the yield points, the sandwich-structured models can break at almost same stress before the yield points in spite of the differences in their Young’s moduli. Thus, there should be a critical interfacial strength above which the stiffness of the polymer significantly affects the tensile strength.

Additionally, our calculations do not consider the breaking of chemical bonds, which can affect failure behaviors. To check the contribution of the bond breaking, we monitored the maximum bond force exerted on C–C bonds in the backbones of the polymers during the tensile tests using the sandwich-structured models. According to a theoretical work using DFT calculations, a force around 6 nN is required to break a C–C bond in alkanes such as ethane and butane.^35^ By examining the maximum bond force during the mechanical tests, there is no clear trend that it increases as a strain rises, and thus it does not reach the breaking force of around 6 nN (see Figure S5 in Supporting Information). This means that the interfaces in our models are not strong for the bonds to be broken. Therefore, even if bond breaking were taken into account in our calculations, the trend would not be significantly changed.

In more general situations, there would be no significant breaking of bonds at the yield points of the polymer between the surfaces because yielding usually occurs by the slippage of polymer chains instead of bond breaking. This means that in polymer–metal joints where the yield stress of the polymer is larger than the interfacial strength, and thus is observed as a tensile strength, bond breaking would not change the tendency that the tensile strength increases with a rise in Young’s modulus. On the other hand, in the joints that provide a larger interfacial strength than yield stress, bond breaking can happen after yielding and before the interfacial strength. If it is taken into account, it can lead to cohesive failures during this period. In other words, as long as interfacial failures are observed, the contribution of bond breaking to the tensile strength may be limited and, therefore, would not significantly affect the tendency that a higher Young’s modulus leads to a higher tensile strength. Additionally, even in the situations where the failure mode changes from an interfacial failure to a cohesive failure by the bond breaking and thus a tensile strength corresponds to an ultimate strength, the Young’s modulus can be still an influential parameter on the tensile strength because ultimate strength is usually affected by Young’s modulus.

Regarding the influences of Young’s modulus on interfacial failure behaviors, although there are few studies on polymer–metal or metal-oxide interfaces, Agrawal and Raj related Young’s modulus to the interfacial shear strength for a ceramic–metal interface by
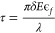
3where τ is the maximum interfacial shear strength observed in their mechanical tests, δ is the thickness of ceramic, *E* is its Young’s modulus, ϵ_*f*_ is the strain when a crack starts to generate, and λ is a space between cracks on the ceramic at a stage when the number of cracks becomes saturated.^36^ In the tensile tests using the sandwich-structured models, the yield strains, which seem to correspond to ϵ_*f*_ in eq 3, are almost the same as in Figure 6 (see Figure S6 in Supporting Information). Therefore, the relationship between Young’s modulus and tensile strength shown in Figure 8 appears consistent with this equation.

## Conclusions

This study focused on the interfaces between iPP or iPP-g-MA and hydroxylated γ-Al_2_O_3_ and examined the influence of Young’s moduli of iPP and iPP-g-MA and the larger interfacial interaction of iPP-g-MA on the interfacial failure behaviors. Our MD calculations reveal that the tensile strength observed in an interfacial failure rises with increasing Young’s modulus. This is because the interfacial failures result from voids forming within iPP or iPP-g-MA matrix and the higher Young’s modulus makes it more difficult for these voids to form. Regarding the contribution of the higher interaction of iPP-g-MA, as Young’s modulus of iPP-g-MA gets larger, it more significantly affects the tensile strength in an interfacial failure. Thus, for iPP-g-MA with a larger Young’s modulus, its higher interfacial interaction works to increase the tensile strength in an interfacial failure.

These results can offer useful design principles to improve interfacial failure behaviors at polymer–metal or metal oxide interfaces. In general, when interfacial failures are observed in mechanical tests using polymer–metal joints, enhancing the interfacial interactions might appear to be the most effective way to improve the strengths. However, our calculations provide another helpful strategy of increasing Young’s modulus of the polymer.

## Supporting Information Available

Validation of the sandwich-structured model of iPP-g-MA, demonstration of the differences between peel and tensile modes, the influence of the method of changing Young’s modulus, the contribution of chemical bond breaking, and the contribution of Young’s modulus to the yield strain observed in the san dwich-structured models (PDF)

## References

[ref1] Lamb W. F.; et al. A Review of Trends and Drivers of Greenhouse Gas Emissions by Sector from 1990 to 2018. Environ. Res. Lett. 2021, 16, 073005 10.1088/1748-9326/abee4e.

[ref2] Kleinbaum S.; Jiang C.; Logan S. Enabling Sustainable Transportation through Joining of Dissimilar Lightweight Materials. MRS Bull. 2019, 44, 608–612. 10.1557/mrs.2019.178.

[ref3] Awaja F.; Gilbert M.; Kelly G.; Fox B.; Pigram P. J. Adhesion of Polymers. Prog. Polym. Sci. 2009, 34, 948–968. 10.1016/j.progpolymsci.2009.04.007.

[ref4] Bukhari M. D.; Gohar G. A.; Akhtar A.; Ullah S.; Akram M.; Abid J.; Raza H. Adhesion Theories and Effect of Surface Roughness on Energy Estimation and Wettability of Polymeric Composites Bonded Joints: A-Review. VW Appl. Sci. 2019, 2, 74–86. 10.36297/vw.applsci.v2i1.37.

[ref5] Grujicic M.; Sellappan V.; Omar M. A.; Seyr N.; Obieglo A.; Erdmann M.; Holzleitner J. An Overview of the Polymer-to-Metal Direct-Adhesion Hybrid Technologies for Load-Bearing Automotive Components. J. Mater. Process. Technol. 2008, 197, 363–373. 10.1016/j.jmatprotec.2007.06.058.

[ref6] Suganuma Y.; Yamamoto S.; Kinjo T.; Mitsuoka T.; Umemoto K. Wettability of Al_2_O_3_ Surface by Organic Molecules: Insights from Molecular Dynamics Simulation. J. Phys. Chem. B 2017, 121, 9929–9935. 10.1021/acs.jpcb.7b07062. 28960991

[ref7] Suganuma Y.; Mitsuoka T.; Yamamoto S.; Kinjo T.; Yoneyama H.; Umemoto K. Wettability of Primer-Treated Al_2_O_3_ Surfaces by Bisphenol A Diglycidyl Ether: Determination of the Mechanism from Molecular Dynamics Simulations and Experiments. J. Phys. Chem. B 2019, 123, 4434–4442. 10.1021/acs.jpcb.9b00680. 31059261

[ref8] Duguet T.; Gavrielides A.; Esvan J.; Mineva T.; Lacaze-Dufaure C. DFT Simulation of XPS Reveals Cu/Epoxy Polymer Interfacial Bonding. J. Phys. Chem. C 2019, 123, 30917–30925. 10.1021/acs.jpcc.9b07772.

[ref9] Prathab B.; Subramanian V.; Aminabhavi T. M. Molecular Dynamics Simulations to Investigate Polymer–Polymer and Polymer–Metal Oxide Interactions. Polymer 2007, 48, 409–416. 10.1016/j.polymer.2006.11.014.

[ref10] Iwamoto N. Molecularly Derived Mesoscale Modeling of an Epoxy/Cu Interface: Interface Roughness. Microelectron. Reliab. 2013, 53, 1101–1110. 10.1016/j.microrel.2013.02.015.

[ref11] Agrawal D. C.; Raj R. Measurement of the Ultimate Shear Strength of a Metal-Ceramic Interface. Acta Metall. 1989, 37, 1265–1270. 10.1016/0001-6160(89)90120-X.

[ref12] Agrawal D. C.; Raj R. Ultimate Shear Strengths of Copper-Silica and Nickel-Silica Interfaces. Mater. Sci. Eng., A 1990, 126, 125–131. 10.1016/0921-5093(90)90118-M.

[ref13] Qu J.; Ouyang L.; Kuo C. C.; Martin D. C. Stiffness, Strength and Adhesion Characterization of Electrochemically Deposited Conjugated Polymer Films. Acta Biomater. 2016, 31, 114–121. 10.1016/j.actbio.2015.11.018. 26607768 PMC4728054

[ref14] Benedetti A.; Fernandes P.; Granja J. L.; Sena-Cruz J.; Azenha M. Influence of Temperature on the Curing of an Epoxy Adhesive and Its Influence on Bond Behaviour of NSM-CFRP Systems. Composites, Part B 2016, 89, 219–229. 10.1016/j.compositesb.2015.11.034.

[ref15] Jain S.; Goossens J.; van Duin M. Synthesis, Characterization and Properties of (Vinyl Triethoxy Silane-grafted PP)/Silica Nanocomposites. Macromol. Symp. 2006, 233, 225–234. 10.1002/masy.200690022.

[ref16] Dal Castel C.; Pelegrini T.; Barbosa R. V.; Liberman S. A.; Mauler R. S. Properties of Silane Grafted Polypropylene/Montmorillonite Nanocomposites. Composites, Part A 2010, 41, 185–191. 10.1016/j.compositesa.2009.09.017.

[ref17] Toro P.; Quijada R.; Peralta R.; Yazdani-Pedram M. Influence of Grafted Polypropylene on the Mechanical Properties of Mineral-Filled Polypropylene Composites. J. Appl. Polym. Sci. 2007, 103, 2343–2350. 10.1002/app.24956.

[ref18] Chen M. A.; Li H. Z.; Zhang X. M. Improvement of Shear Strength of Aluminium-Polypropylene Lap Joints by Grafting Maleic Anhydride onto Polypropylene. Int. J. Adhes. Adhes. 2007, 27, 175–187. 10.1016/j.ijadhadh.2006.01.008.

[ref19] Kim H. S.; Lee B. H.; Choi S. W.; Kim S.; Kim H. J. The Effect of Types of Maleic Anhydride-Grafted Polypropylene (MAPP) on the Interfacial Adhesion Properties of Bio-Flour-Filled Polypropylene Composites. Composites, Part A 2007, 38, 1473–1482. 10.1016/j.compositesa.2007.01.004.

[ref20] Astigarraga V.; Gondra K.; Valea Á.; Pardo Aurrekoetxea G. Improvement of Adhesive Bonding of Polypropylene and Maleic Anhydride Grafted Polypropylene Blends to Aluminium by Means of Addition of Cyclic Butylene Terephthalate. J. Adhes. 2019, 95, 286–307. 10.1080/00218464.2018.1437415.

[ref21] Meunier M. Introduction to Materials Studio. EPJ Web Conf. 2012, 30, 04001 10.1051/epjconf/20123004001.

[ref22] Quynn R. G.; Riley J. L.; Young D. A.; Noether H. D. Density, Crystallinity, and Heptane Insolubility in Isotactic Polypropylene. J. Appl. Polym. Sci. 1959, 2, 166–173. 10.1002/app.1959.070020506.

[ref23] Plimpton S. Fast Parallel Algorithms for Short-Range Molecular Dynamics. J. Comput. Phys. 1995, 117, 1–19. 10.1006/jcph.1995.1039.

[ref24] aNosé S. A Molecular Dynamics Method for Simulations in the Canonical Ensemble. Mol. Phys. 1984, 52, 255–268. 10.1080/00268978400101201.

[ref25] aSun H. Field for Computation of Conformational Energies, Structures, and Vibrational Frequencies of Aromatic Polyesters. J. Comput. Chem. 1994, 15, 752–768. 10.1002/jcc.540150708.

[ref26] Knaup J. M.; Köhler C.; Frauenheim T.; Blumenau A. T.; Amkreutz M.; Schiffels P.; Schneider B.; Hennemann O. D. Computational Studies on Polymer Adhesion at the Surface of γ-Al_2_O_3_. I. the Adsorption of Adhesive Component Molecules from the Gas Phase. J. Phys. Chem. B 2006, 110, 20460–20468. 10.1021/jp063814w. 17034231

[ref27] Aykol M.; Persson K. A. Oxidation Protection with Amorphous Surface Oxides: Thermodynamic Insights from Ab Initio Simulations on Aluminum. ACS Appl. Mater. Interfaces 2018, 10, 3039–3045. 10.1021/acsami.7b14868. 29297220

[ref28] Tan X.; Ren X.; Li J.; Wang X. Theoretical Investigation of Uranyl Ion Adsorption on Hydroxylated γ-Al_2_O_3_ Surfaces. RSC Adv. 2013, 3, 19551–19559. 10.1039/c3ra42853b.

[ref29] Ionescu A.; Allouche A.; Aycard J. P.; Rajzmann M.; Hutschka F. Study of γ-Alumina Surface Reactivity: Adsorption of Water and Hydrogen Sulfide on Octahedral Aluminum Sites. J. Phys. Chem. B 2002, 106, 9359–9366. 10.1021/jp020145n.

[ref30] Digne M.; Sautet P.; Raybaud P.; Euzen P.; Toulhoat H. Use of DFT to Achieve a Rational Understanding of Acid-Basic Properties of γ-Alumina Surfaces. J. Catal. 2004, 226, 54–68. 10.1016/j.jcat.2004.04.020.

[ref31] Hirshfeld F. L. Bonded-Atom Fragments for Describing Molecular Charge Densities. Theor. Chim. Acta 1977, 44, 129–138. 10.1007/BF00549096.

[ref32] Segall M. D.; Lindan P. J.; Probert M. J.; Pickard C. J.; Hasnip P. J.; Clark S. J.; Payne M. C. First-Principles Simulation: Ideas, Illustrations and the CASTEP Code. J. Phys.: Condens. Matter 2002, 14, 2717–2744. 10.1088/0953-8984/14/11/301.

[ref33] Clark S. J.; Segall M. D.; Pickard C. J.; Hasnip P. J.; Probert M. I.; Refson K.; Payne M. C. First Principles Methods Using CASTEP. Z. Krist. 2005, 220, 567–570. 10.1524/zkri.220.5.567.65075.

[ref34] Perdew J. P.; Burke K.; Ernzerhof M. Generalized Gradient Approximation Made Simple. Phys. Rev. Lett. 1996, 77, 3865–3868. 10.1103/PhysRevLett.77.3865. 10062328

[ref35] Monkhorst H. J.; Pack J. D. Special Points for Brillouin-Zone Integrations. Phys. Rev. B 1976, 13, 5188–5192. 10.1103/PhysRevB.13.5188.

[ref36] Zuo F.; Keum J. K.; Chen X.; Hsiao B. S.; Chen H.; Lai S. Y.; Wevers R.; Li J. The Role of Interlamellar Chain Entanglement in Deformation-Induced Structure Changes during Uniaxial Stretching of Isotactic Polypropylene. Polymer 2007, 48, 6867–6880. 10.1016/j.polymer.2007.08.065.

[ref37] Stern C.; Frick A.; Weickert G. Relationship between the Structure and Mechanical Properties of Polypropylene: Effects of the Molecular Weight and Shear-Induced Structure. J. Appl. Polym. Sci. 2007, 103, 519–533. 10.1002/app.24156.

[ref38] Khonakdar H. A.; Jafari S. H.; Wagenknecht U.; Jehnichen D. Effect of Electron-Irradiation on Cross-Link Density and Crystalline Structure of Low- and High-Density Polyethylene. Radiat. Phys. Chem. 2006, 75, 78–86. 10.1016/j.radphyschem.2005.05.014.

[ref39] Yuan X.; Wang J.; Wang C.; Gao S.; Guo S.; Zhang Y. Influence of 1,2-Polybutadiene on Properties of Dicumyl Peroxide Cured Brominated Butyl Rubber. J. Appl. Polym. Sci. 2016, 133, 43280 10.1002/app.43280.

[ref40] Yamamoto S.; Kuwahara R.; Aoki M.; Shundo A.; Tanaka K. Molecular Events for an Epoxy-Amine System at a Copper Interface. ACS Appl. Polym. Mater. 2020, 2, 1474–1481. 10.1021/acsapm.9b01154.

[ref41] Solano Canchaya J. G.; Dequidt A.; Garruchet S.; Latour B.; Martzel N.; Devémy J.; Goujon F.; Blaak R.; Schnell B.; Munch E.; Seeboth N.; Malfreyt P. Development of a Coarse-Grain Model for the Description of the Metal Oxide-Polymer Interface from a Bottom-up Approach. J. Chem. Phys. 2019, 151, 64703 10.1063/1.5115148.

[ref42] Roberts D. R.; Holder S. J. Mechanochromic Systems for the Detection of Stress, Strain and Deformation in Polymeric Materials. J. Mater. Chem. 2011, 21, 8256–8268. 10.1039/c0jm04237d.

[ref43] Hillborg H.; Sandelin M.; Gedde U. W. Hydrophobic Recovery of Polydimethylsiloxane after Exposure to Partial Discharges as a Function of Crosslink Density. Polymer 2001, 42, 7349–7362. 10.1016/S0032-3861(01)00202-6.

[ref44] Chen M. A.; Li H. Z.; Zhang X. M. Improvement of Shear Strength of Aluminium-Polypropylene Lap Joints by Grafting Maleic Anhydride onto Polypropylene. Int. J. Adhes. Adhes. 2007, 27, 175–187. 10.1016/j.ijadhadh.2006.01.008.

[ref45] Chen M.-A.; Zhang X.-M.; Huang R.; Lu X.-B. Mechanism of Adhesion Promotion between Aluminium Sheet and Polypropylene with Maleic Anhydride-Grafted Polypropylene by γ-Aminopropyltriethoxy Silane. Surf. Interface Anal. 2008, 40, 1209–1218. 10.1002/sia.2871.

