# Supplementary material for: Effect of Varying Stiffness and Functionalization on the Interfacial Failure Behavior of Isotactic Polypropylene on Hydroxylated γ-Al2O3 by MD Simulation
Source: ACS Appl Mater Interfaces. 2023 Jan 20;15(4):6133–41. doi: 10.1021/acsami.2c19593 (PMC9906630; doi:10.1021/acsami.2c19593)
Supplement: Supplementary file 1 — am2c19593_si_001.pdf [file am2c19593_si_001.pdf]

Supporting Information

Effect of Varying Stiffness and Functionalization on the Interfacial Failure Behavior of Isotactic Polypropylene on Hydroxylated γ-Al_2O_3 by MD Simulation

Yoshitake Suganuma* and James A. Elliott*

Department of Materials Science and Metallurgy, University of Cambridge, 27 Charles Babbage Rd, Cambridge CB3 0FS

E-mail: ys519@cam.ac.uk; jae1001@cam.ac.uk

Validation of Sandwich-Structured Model of iPP-g-MA

To validate the sandwich-structured model of iPP-g-MA before the entanglement points are introduced, the profiles of the density and orientational order parameters were evaluated in the same way as the paper (Figure S1). These graphs shows the same features reported in the sandwich-structured model of iPP. The molecules are absorbed on the surfaces, which leads to the peaks in the density profile, in-plane alignment of the backbone, and normal alignment of the side groups at the interfaces in the profile of orientational order parameter. These interfacial structures are in a good agreement with those observed in the sandwich-structured model of iPP and other computational works on interfaces between a polymer and a metal or metal oxide surface.[1,2]

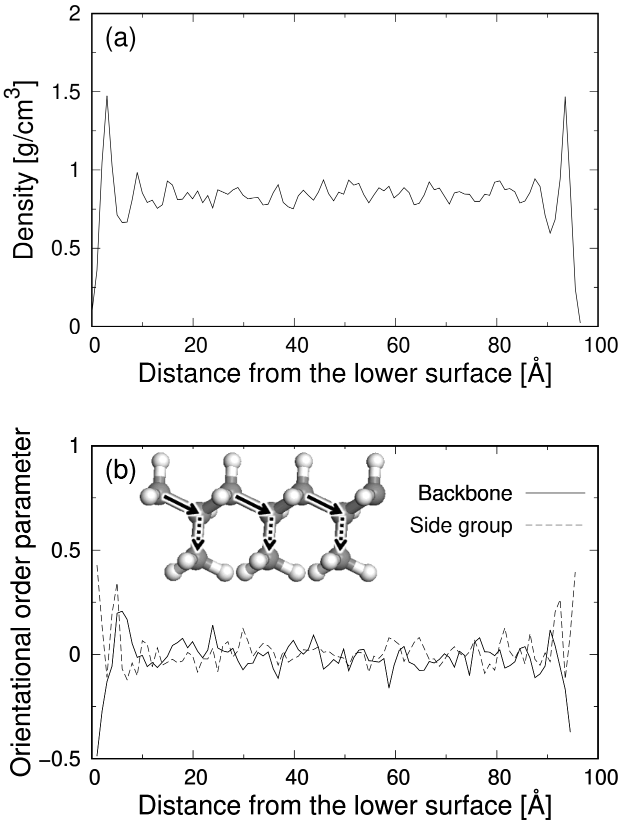

Figure S1: The profiles of (a) the density of iPP-g-MA and (b) orientational order parameters as a function of the distance from the lower surface in the sandwich-structured model of iPP-g-MA.

Differences in Failure Behaviors between in Peel and Tensile Modes

To make clearer the difference in the contribution of MA groups in iPP-g-MA between in peel and tensile modes, one molecule of iPP or iPP-g-MA was placed on the surface, and, then, detached from the surface in these failure modes while the stress exerted on the surface was recorded. First, one iPP or iPP-g-MA molecule was placed on the hydroxylated γ-Al_2O_3 surface with the dimension of around 100×100 Å^2. The molecules consist of 50 repeat units, and the iPP-g-MA molecule was grafted with three MA groups. Then, the molecules on the surface was subjected to a MD simulaiton for 500 ps to be relaxed.

Next, from the last snapshots in the MD simulations, the molecules were removed from the surface in peel and tensile modes. In a peel mode, a carbon atom at the edge of iPP or

iPP-g-MA backbone was moved along the z-axis with a constant speed of 1.1×10^{-3} Å/fs, which is almost the same rate as used in MD simulations to obtain stress-strain curves of sandwich-structured models in the paper. On the other hand, in a tensile mode, one carbon atom every 2 repeat units, i.e. 25 carbon atoms in total, which is the same frequency as iPP-EP1/2 and iPP-g-MA-EP1/2, was moved along the z-axis with the same rate. Then, the z-components of the forces exerted on the atoms of the surface were summed and divided by the surface area to obtain the stress on the surface.

Figure S2 shows the stress as a function of the displacement of the moved atoms. In a peel mode, the stress of iPP-g-MA is not significantly different from that of iPP. Meanwhile, in a tensile mode, the maximum stress of iPP-g-MA is clearly larger than that of iPP. This indicates that MA groups affect the interfacial strengths more significantly in a tensile mode than in a peel mode. This is because that the three MA groups are peeled one by one in a peel mode, while they are pulled off at the same time in a tensile mode. This is why, as Young's modulus of iPP-g-MA gets larger, MA groups work more effectively to improve the interfacial strength.

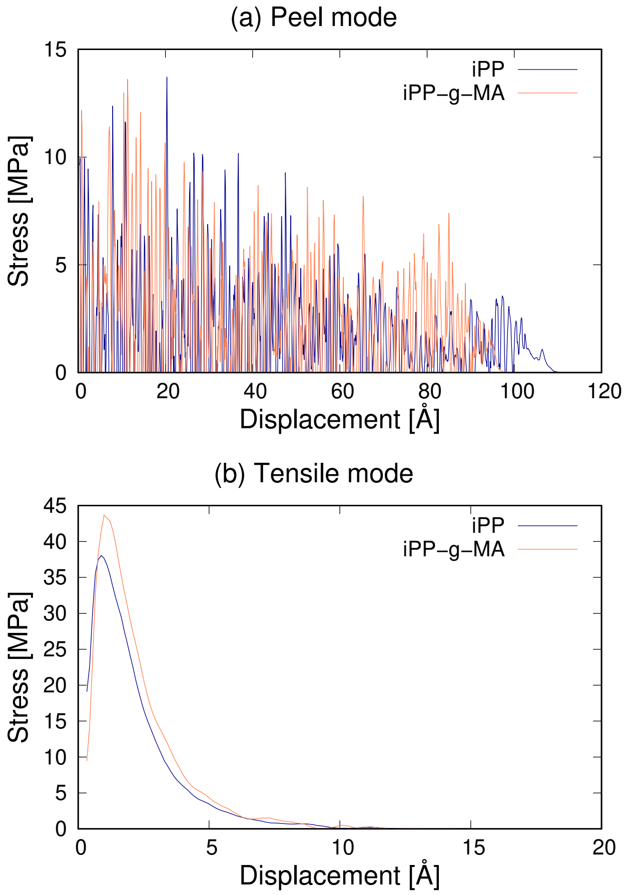

Figure S2: The stress exerted on the surface as a function of the displacement of pulled atoms when iPP or iPP-g-MA is pulled in a (a) peel mode and (b) tensile mode.

Influence of Method to Increase Young's Modulus

To confirm the influence of the method to change Young's modulus, we prepared structures of iPP with different Young's moduli by increasing the interaction parameters for iPP instead of by introducing the entanglement points. We increased the ϵ values in the Lennard-Jones potential for the non-bonded interactions between atoms in iPP to 2.5, 5, and 10 times the original values. Then, we obtained the stress-strain curves of the bulk structures of iPP with increased interaction parameters and their sandwich-structured models. Figure S3 shows their stress-strain curves.

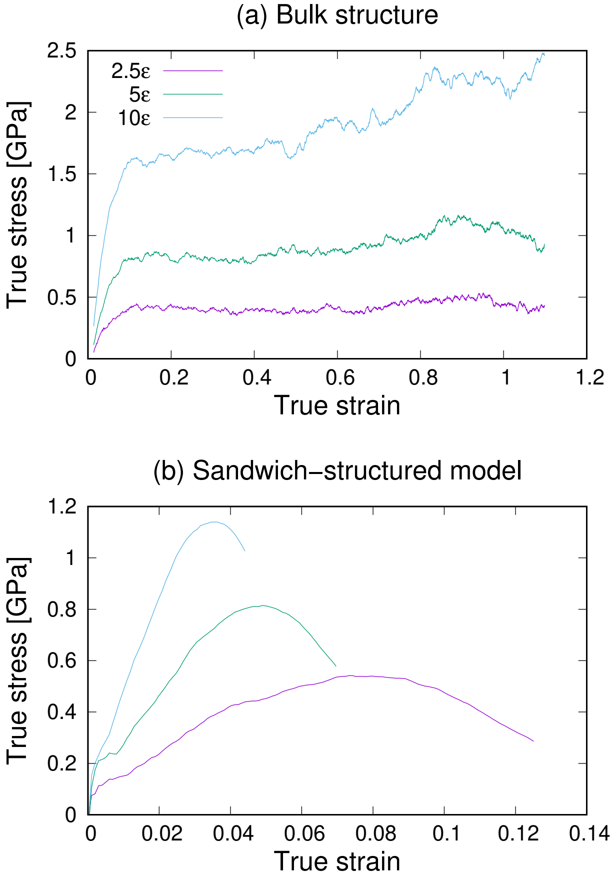

Figure S3: The stress-strain curves of the (a) bulk structures of iPP with increased interaction parameters and (b) their sandwich-structured models.

Next, Young's moduli of the bulk structures and the tensile strengths were evaluated from Figures S3(a) and (b) respectively. In all of the sandwich-structured models in Figure S3, the tensile strength was observed when an interface failed, which means that the tensile strength corresponds to the interfacial strength. Figure S4 shows the relationship between Young's modulus and tensile strength obtained by the increasing the interaction parameters compared to the method of introduction of the entanglement points. Although the best-fit lines are not completely identical with each other, they demonstrate the same tendency that the tensile strength increases as Young's modulus increases. Thus, although the method to change the stiffness might result in different contribution of Young's modulus to the tensile strength, it remains an appropriate parameter affecting the tensile strength, independent of

the network structure of polymer.

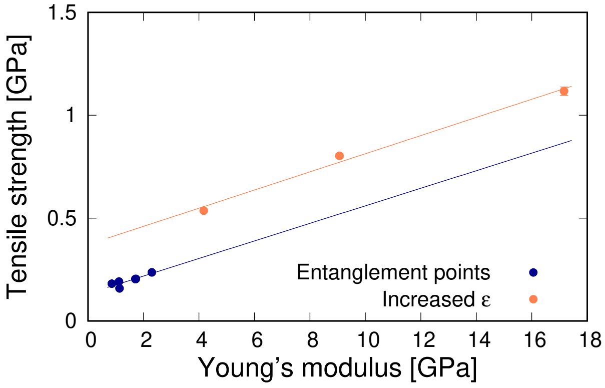

Figure S4: The relationship between Young's modulus and tensile strength obtained by the increased ϵ values and by the introduction of the entanglement points.

Contribution of Chemical Bond Breaking

To check the possible contribution of bond breaking to the failure behavior observed in the sandwich-structured models, we monitored the maximum bond force exerted on C-C bonds in the backbones of the polymers during the tensile tests using the sandwich-structured models (Figure S5). According to theoretical work using DFT calculations, a force of around 6 nN is required to break a C-C bond in alkanes such as ethane and butane.[3] Figure S5 demonstrates that there is no clear trend for the maximum bond force to increase as strain rises, and thus it does not reach the breaking force of around 6 nN. This means that the interfaces in our models are not sufficiently strong for the bonds to be broken. Therefore, if bond breaking is taken into account in our calculations, the trend will not be significantly changed.

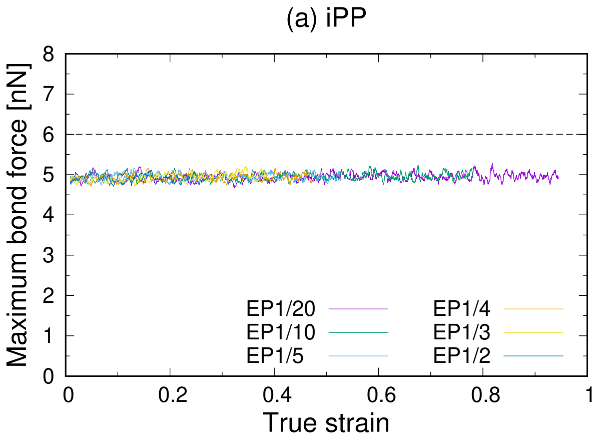

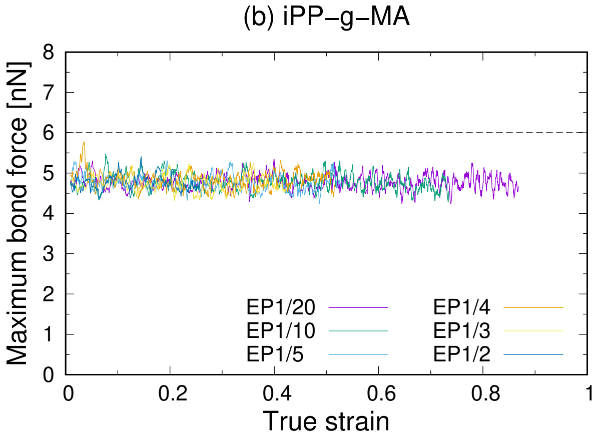

Figure S5: The maximum bond force exerted on C-C bonds in the backbones of (a) iPP and (b) iPP-g-MA during the mechanical tests using the sandwich-structured models.

The Yield Strain of Sandwich-Structured Models.

The yield strains observed in the stress-strain curves of the sandwich-structured models were evaluated to ensure that our calculations follow Eq. (3) in the paper. The procedure to determine the yield points is described in the paper. Figure S6 shows the yield strain of the sandwich-structured models as a function of Young's modulus of the amorphous structures of iPP or iPP-g-MA. This indicates that the strain values are almost the same and independent of Young's moduli. Therefore, our calculations appear to be in a good agreement with Eq. (3) in the paper.

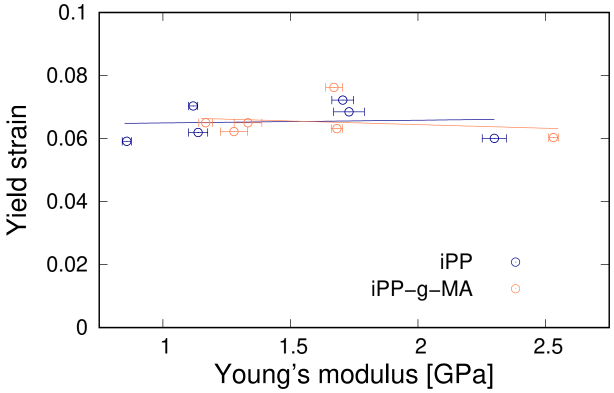

Figure S6: The yield strain of the sandwich-structured models of iPPs and iPP-g-MAs as a function of Young's modulus.

References

(1) Yamamoto, S.; Kuwahara, R.; Aoki, M.; Shundo, A.; Tanaka, K. Molecular Events for an EpoxyAmine System at a Copper Interface. *Cite This ACS Appl. Polym. Mater* **2020**, *2020*, 1474–1481.

(2) Solano Canchaya, J. G.; Dequidt, A.; Garruchet, S.; Latour, B.; Martzel, N.; Devémy, J.; Goujon, F.; Blaak, R.; Schnell, B.; Munch, E.; Seeboth, N.; Malfreyt, P. Development of a Coarse-grain Model for the Description of the Metal Oxide-Polymer Interface from a Bottom-Up Approach. *J. Chem. Phys.* **2019**, *151*, 64703.

(3) Tolladay, M.; Scarpa, F.; Allan, N. L. Interatomic Forces Breaking Carbon-Carbon Bonds. *Carbon N. Y.* **2021**, *175*, 420–428.
